# Assessment of glucose and lipid metabolism in patients with polycystic ovary syndrome with and without Hashimoto’s thyroiditis

**DOI:** 10.1097/MD.0000000000033205

**Published:** 2023-03-17

**Authors:** Cenlin Jia, Lin Zhang, Wenhua Liu, Xiangyan Zhang, Hongyan Wu

**Affiliations:** a Department of Gynecology, Jinhua Municipal Central Hospital, Jinhua, China; b Department of Gynecological Endocrinology, Hangzhou Women’s Hospital, Hangzhou, China.

**Keywords:** glucose, Hashimoto’s thyroiditis, lipid metabolism, polycystic ovary syndrome

## Abstract

To investigate glucose and lipid metabolism in patients with polycystic ovary syndrome (PCOS) with and without Hashimoto’s thyroiditis (HT). In the present study, 103 women were included as controls and a total of 213 patients (49 patients with HT and 164 patients without HT) diagnosed with PCOS. The oral glucose tolerance, insulin release, thyroid function, and lipid levels were measured. PCOS patients had significantly higher levels of fasting insulin (FINS), hemostasis of model assessment-insulin resistance, low-lipoprotein cholesterol, triglyceride, apolipoprotein B, apolipoprotein B/apolipoprotein A1, and homocysteine than the controls. PCOS Patients with HT + had higher FINS, 60FINS, 120FINS, and insulin resistance levels than those without Hashimoto’s thyroiditis group. HT + group had higher total cholesterol, and thyroid-stimulating hormone levels, while free triiodothyronine, and free thyroxine levels were significantly lower. PCOS can lead to disorders of glucolipid metabolism, PCOS with Hashimoto’s thyroiditis may further exacerbate disorders of glucose and lipid metabolism, and therefore thyroid function assessment in patients with PCOS needs to be emphasized.

## 1. Introduction

Polycystic ovary syndrome (PCOS) is the most common gynecological endocrine disease in the reproductive age of women, with an incidence of 5% to 10% in European and American countries, and the prevalence rate in China is 5.61%.^[1–3]^ The thyroid hormone plays an important role not only in endocrine and metabolic regulation but also in reproductive health. Thyroid receptors and thyroid-stimulating hormone (TSH) receptors are expressed in the ovary and uterus. Thyroid deficiency may affect gonadal function and fertility, leading to delayed onset of puberty and anovulatory cycle.^[4]^ Hashimoto’s thyroiditis (HT) is characterized by an elevated concentration of antithyroid antibodies, frequent thyroid dysfunctions in varying degrees, and a typical hypoechogenic pattern on thyroid ultrasound due to lymphocytic infiltration and eventual fibrous of the thyroid. Patients with PCOS present low levels of progesterone and high or normal levels of estradiol because of oligo/anovulation. Estradiol and estradiol/progesterone ratio were positively correlated with anti-TPO levels in PCOS women. The imbalance between estrogen and progesterone levels may be responsible for the frequent co-occurrence of autoimmune thyroiditis in women with PCOS.^[5]^ Numerous studies have proved that thyroid diseases, especially HT, are closely related to PCOS,^[6]^ and the incidence rate of Hashimoto’s thyroiditis in PCOS is 3 times that of ordinary women.^[7]^ Patients with PCOS demonstrate an increased risk for metabolic disorders including obesity, insulin resistance, type 2 diabetes mellitus, dyslipidemia, hypertension, and cardiovascular disease.^[8,9]^ Furthermore, patients with PCOS in combination with HT show more severe metabolic symptoms than those with PCOS or HT alone.^[10]^ Therefore, it is particularly important to study the glucose and lipid metabolism in PCOS patients with HT. This study aims to investigate the effect of Hashimoto’s thyroiditis on glucose and lipid metabolism in patients with polycystic ovary syndrome.

## 2. Subjects and Methods

### 2.1. Data source and collection

This cross-sectional study enrolled 213 PCOS patients, including 49 patients with Hashimoto’s thyroiditis (HT + ) and 164 patients without Hashimoto’s thyroiditis (HT−), who was admitted to the outpatient of Jinhua Municipal Central Hospital from September 2018 to February 2022. All enrolled cases were aged between 16 and 40 years. We randomly selected 103 women in the population as controls, 15 of whom with HT + . The study was reviewed and approved by the hospital ethics committee, and all subjects signed informed consent. The software program of Power and Sample Size (PASS15.0.5, NCSS Inc, Kaysville, UT) was used to calculate the sample size.^[11]^

The Rotterdam criteria were used for the diagnosis of PCOS: the presence of 2 of the following 3 criteria, namely: Oligomenorrhea (cycles lasting longer than 35 days) or amenorrhea (<2 menstrual cycles in the past 6 months); Clinical manifesttions of hyperandrogenism or hyperandrogenemia; Ultrasound showed polycystic ovary and other diseases that could cause hyperandrogenic diseases and abnormal ovulation were excluded. At least at present 2 of the above criteria can be diagnosed as PCOS.

#### 1.2.1. Exclusion criteria.

1. With-non classic congenital adrenal hyperplasia, hyperprolactinemia, thyroid organic diseases, Cushing’s syndrome, ovarian or adrenal gland secretion of androgen tumors, liver and kidney dysfunction, diabetes, hypertension, and other diseases.
2. No hormone drugs, promoting drugs, and androgen lowering drugs in recent 3 months.

Hashimoto’s thyroiditis is diagnosed when anti-thyroglobulin and antithyroid peroxidase antibodies are positive and the color doppler of the thyroid gland is compatible with the imaging abnormalities of Hashimoto’s thyroiditis.

### 2.2. Determination of glucose and lipid metabolism

Fasting venous blood was collected from 8:00 am to 10:00 am from all subjects, and all PCOS women have an oral glucose tolerance test and insulin release (IR) test with a 75 g glucose load. Blood samples were measured for serum glucose and insulin at 0, 60, and 120 minutes. Serum levels of fasting plasma glucose, fasting insulin (FINS), 60INS, 120INS, total cholesterol (TC), triglyceride (TG), low-lipoprotein cholesterol (LDL), high-density lipoprotein cholesterol (HDL), apolipoprotein A1 (apoA1), apolipoprotein B (apo B), and homocysteine (HCY) were measured by Beckman Coulter AU5821 biochemistry analyzer. Insulin resistance was calculated by the homeostasis model assessment-insulin resistance (HOMA-IR) [fasting plasma glucose (mmol/L) × fasting serum insulin (μIU/mL)/22.5].

### 2.3. Determination of thyroid function

TSH, total triiodothyronine, total thyroxine, free triiodothyronine, free thyroxine, thyroid peroxidase antibody (TPO-Ab), and anti-thyroglobulin antibody (TG-Ab) were measured using the DXI800 immunoassay analyzer from Beckman Coulter, with TG-Ab -Ab > 60 IU/mL and (or) TPO-Ab > 60 IU/mL is positive.

### 2.4. Statistical analysis

Summary statistics are presented as the mean ± SD for continuous variables. The Kolmogorov-Smirnov test was used to test the normal distribution of variables. Mann–Whitney *U* test was used in the analysis of the data that did not conform to the normal distribution between the measurement values of the 2 groups, and the Student *t* test was used in the analysis of the data with normal distribution. The Pearson chi-square test for categorical variables. Pearson correlation was used to determine the relationship between each variable and TG-Ab/TPO-Ab. The SPSS 19.0 software was used for all statistical analyses. *P* values < s.05 was determined statistically significant.

## 3. Results

Compared to the normal population, PCOS patients had significantly higher levels of FINS, HOMA-IR, LDL, TG, apolipoprotein B (apoB), apoB/apoA1, and HCY, apoA1 was also significantly lower in PCOS patients, and the prevalence of Hashimoto’s thyroiditis was 3 times as high in PCOS patients as in the correct population (Table 1). In the comparison between the HT + and HT− groups, no significant differences were identified in age, height, weight, and BMI. The levels of FINS, 60INS, 120INS, and HOMA-IR were considerably greater in HT + than in HT−. The TC level was much higher in the HT + group, while there were no substantial differences in TG, LDL, HDL, apoA1, apo B, and HCY between the 2 groups. The levels of free thyroxine and free triiodothyronine were much lower in the HT + group, whereas TSH was significantly higher (Table 2). The results of correlation analysis showed that TG-Ab/TPO-Ab was positively correlated with FINS, 60 INS (*P* < .05), and positively correlated with 120 INS, IR and TC but not statistically different (*P* > .05) (Table 3).

**Table 1:** Comparison of parameters between controls with polycystic ovary syndrome patients.

	Controls (n = 103, mean ± SD)	PCOS (n = 213, mean ± SD)	*P* value
Age (yr)	27.63 ± 2.67	27.30 ± 4.90	.52
Height (cm)	160.57 ± 4.61	161.23 ± 4.99	.25
Weight (kg)	57.14 ± 7.62	59.05 ± 10.64	.37
BMI (kg/m2)	22.21 ± 3.15	22.70 ± 3.91	.26
FPG (mmol/L)	4.75 ± 1.40	4.67 ± 0.62	.45
FINS (uIU/mL)	7.91 ± 1.29	11.40 ± 6.28	.00*
HOMA-IR	1.67 ± 0.59	2.40 ± 1.30	.00*
HDL(mmol/L)	1.52 ± 0.31	1.45 ± 0.49	.20
LDL (mmol/L)	2.38 ± 0.54	2.73 ± 0.74	.00*
TC (mmol/L)	4.55 ± 0.79	4.50 ± 1.20	.67
TG (mmol/L)	0.98 ± 0.46	1.33 ± 1.01	.00*
apoA1 (g/L)	1.48 ± 0.23	1.29 ± 0.25	.00*
apoB (g/L)	0.70 ± 0.19	1.05 ± 0.30	.00*
apoB/apoA1	0.49 ± 0.19	0.85 ± 0.30	.00*
HCY (umol/L)	9.38 ± 2.37	10.31 ± 4.24	.04*
T3 (ug/L)	1.66 ± 0.33	1.72 ± 0.59	.52
T4 (ug/L)	11.72 ± 1.47	12.08 ± 2.12	.05*
FT3 (ug/L)	5.27 ± 0.63	5.25 ± 0.80	.58
FT4 (ug/L)	106.99 ± 14.71	107.16 ± 16.83	.21
TSH (mIU/L)	2..15 ± 1.11	2.41 ± 1.46	.39
TG-Ab [n(%)]	9 (8.74%)	43 (20.19%)	.01*
TPO-Ab [n(%)]	8 (7.76%)	36 (16.90%)	.03*

apoA1 = apolipoprotein A1, apoB = apolipoprotein B, BMI = body mass index, FINS = fasting insulin, FPG = fasting plasma glucose, FT3 = free triiodothyronine, FT4 = free thyroxine, HCY = homocysteine, HDL = high-densitylipoprotein, HOMA-IR = homeostatic model assessment index of insulin resistance, LDL = low-density lipoprotein, PCOS = polycystic ovary syndrome, T3 = total triiodothyronine, T4 = total thyroxine, TC = total cholesterol, TG = triglyceride, TG-Ab = anti-thyroglobulin antibody, TPO-Ab = thyroid peroxidase antibody, TSH = thyroid-stimulating hormone.

* Statistically significant difference.

**Table 2:** Comparison of parameters between patients with polycystic ovary syndrome with and without Hashimoto’s thyroiditis.

	Hashimoto’s thyroiditis (n = 49, Mean ± SD)	Without Hashimoto’s thyroiditis (n = 164, Mean ± SD)	*P* value
Age (yr)	27.49 ± 4.17	27.24 ± 5.11	.726
Height (cm)	160.66 ± 5.49	161.40 ± 4.84	.13
Weight (kg)	59.44 ± 12.39	58.93 ± 10.10	.32
BMI (kg/m2)	23.00 ± 4.53	22.62 ± 3.72	.59
FPG (mmol/L)	4.63 ± 0.69	4.67 ± 0.59	.65
FINS (uIU/mL)	13.43 ± 6.07	11.09 ± 6.03	.02*
60INS (uIU/mL)	109.90 ± 63.65	85.04 ± 54.74	.01*
120INS (uIU/mL)	90.30 ± 60.79	67.56 ± 49.61	.01*
HOMA-IR	2.76 ± 1.29	2.30 ± 1.28	.02*
HDL (mmol/L)	1.50 ± 0.40	1.43 ± 0.52	.38
LDL (mmol/L)	2.71 ± 0.65	2.75 ± 0.77	.74
TC (mmol/L)	4.88 ± 1.31	4.38 ± 1.14	.01*
TG (mmol/L)	1.28 ± 0.83	1.34 ± 1.05	.69
apoA1 (g/L)	1.32 ± 0.27	1.29 ± 0.25	.36
apoB (g/L)	1.08 ± 0.31	1.04 ± 0.30	.42
apoB/apoA1	0.86 ± 0.34	0.84 ± 0.29	.68
HCY (umol/L)	10.65 ± 4.38	10.21 ± 4.20	.52
T3 (ug/L)	1.66 ± 0.89	1.72 ± 0.45	.28
T4 (ug/L)	101.95 ± 15.23	107.48 ± 17.18	.86
FT3 (ug/L)	5.04 ± 0.74	5.32 ± 0.79	.03*
FT4 (ug/L)	11.44 ± 1.56	12.22 ± 2.21	.04*
TSH (mIU/L)	2.81 ± 1.28	2.29 ± 1.45	.00*
TG-Ab (IU/mL)	130.20 ± 127.70	30.93 ± 17.63	.00*
TPO-Ab (IU/mL)	196.84 ± 271.31	29.52 ± 16.37	.00*

apoA1 = apolipoprotein A1, apoB = apolipoprotein B, BMI = body mass index, FINS = fasting insulin, FPG = fasting plasma glucose, FT3 = free triiodothyronine, FT4 = free thyroxine, HCY = homocysteine, HDL = high-densitylipoprotein, HOMA-IR = homeostatic model assessment index of insulin resistance, LDL = low-density lipoprotein, T3 = total triiodothyronine, T4 = total thyroxine, TC = total cholesterol, TG = triglyceride, TG-Ab = anti-thyroglobulin antibody, TPO-Ab = thyroid peroxidase antibody, TSH = thyroid-stimulating hormone.

* Statistically significant difference.

**Table 3:** The correlation between TG-Ab/TPO-Ab and the other parameters in HT + group.

		FINS	60INS	120INS	HOMA-IR	TC
TG-Ab	r	0.32	0.29	O.20	0.24	0.30
	*P* value	.03*	.046*	.18	.09	.08
TPO-Ab	r	0.36	0.34	0.26	0.26	0.18
	*P* value	.01*	.02*	.08	.07	.22
T3	r	0.25	0.05	0.09	0.30	0.05
	*P* value	.15	.77	.60	.07	.77
FT3	r	0.21	0.29	0.09	0.19	0.06
	*P* value	.22	.09	.59	.26	.72
T4	r	0.04	0.08	0.04	0.32	0.07
	*P* value	.82	.65	.84	.85	.67
FT4	r	0.00	0.05	0.08	0.03	0.20
	*P* value	.99	.77	.66	.87	.24
TSH	r	0.05	0.05	0.09	0.01	0.12
	*P* value	.79	.77	.61	.94	.49

FINS = fasting insulin, r = correlation coefficient, TC = total cholesterol, TG-Ab = anti-thyroglobulin antibody, TPO-Ab = thyroid peroxidase antibody, TSH = thyroid-stimulating hormone.

* Statistically significant difference.

## 4. Discussion

Polycystic ovary syndrome (PCOS) is an endocrine disorder that occurs mainly in women of reproductive age and is characterized by excessive androgen production, sporadic ovulation, or anovulation. Patients with PCOS present low levels of progesterone and high or normal levels of estradiol because of oligo/anovulation. Estradiol and estradiol/progesterone ratio were positively correlated with anti-TPO levels in PCOS women. The imbalance between estrogen and progesterone levels may be responsible for the frequent co-occurrence of autoimmune thyroiditis in women with PCOS.^[5]^ Our investigation confirmed that PCOS patients are more likely than the general population to have problems with glucolipid metabolism by finding higher levels of FINS, HOMA-IR, LDL, TG, apoB, apoB/apoA1, and HCY in PCOS patients.

Thyroid hormones are also vital for metabolism regulation. Numerous studies have shown that those who have PCOS and HT together have more severe metabolic symptoms than those who have PCOS or HT alone.^[10]^ Polycystic ovarian syndrome (PCOS) and Hashimoto’s thyroiditis (HT) are closely linked,^[12]^ in our study, PCOS patients were 3 times as high as the rate for the general population. Although thyroid disease and PCOS are among the most common endocrine disorders, the pathophysiologic pathways linking the 2 disorders are not well defined and may be related to genetics and metabolism.^[13]^ In PCOS patients, the immunological dysfunction produced by the raised estrogen-to-progesterone ratio may be linked to the increased occurrence of thyroid autoimmune disorders. Estrogen stimulates the immune system by increasing the release of IL-4 in Th2 lymphocytes, IL-1 in monocytes, IL-6 in T lymphocytes, and interferon-Y in Th1 cells. As a natural immunosuppressant, progestin prevents estrogen from stimulating the immune system. However, because progesterone levels are low in PCOS patients due to intermittent ovulation or anovulation, estrogen may overstimulate the immune system, leading to the formation of thyroid autoantibodies.^[14]^

Our study showed that the most significant association between PCOS and HT was an increased metabolic risk of insulin resistance and dyslipidemia. FINS, 60FINS, 120FINS, and IR were significantly higher in patients with polycystic ovary syndrome combined HT + than in patients HT−. Further correlation analysis showed that TG-Ab/TPO-Ab levels were positively correlated with FINS and 60 INS, and the difference was statistically significant. The difference in glucolipid metabolism between the HT + and HT− groups was most likely brought on by TPO-Ab/TG-Ab since there was no discernible correlation between thyroid function and glucolipid metabolism indicators in the correlation study. Conversely, other researchers have found that hypothyroidism in PCOS individuals results in lipid alterations and insulin resistance.^[15]^ Multiple meta-analyses have shown that PCOS patients with combined Hashimoto’s thyroiditis are associated with increased metabolic disturbances, particularly dyslipidemia, affecting triglyceride, LDL, HDL, and total cholesterol levels.^[16]^ This is slightly different from the results of our study, which found a significant increase in TC in patients with PCOS combined with Hashimoto’s thyroiditis compared to those in PCOS alone, and no significant differences were found in LDL, HDL, and triglycerides between the 2 groups. Correlation analysis showed a positive correlation between TG-Ab/TPO-Ab levels and TC, but the difference was not statistically significant. According to Yi Lei, Hashimoto’s thyroiditis affects normal thyroid function and produces hypothyroidism in patients, resulting in lower LDL cholesterol clearance and inhibition of lipoprotein lipase activity, ultimately leading to higher serum TC levels.^[17]^ Patients with PCOS and autoimmune thyroid disease had considerably increased incidences of diabetes, hyperlipidemia, and cardiovascular disease, according to Chun-Wei Ho et al.^[18]^

There were some limitations to this study. A cause–effect relation could not be established since it was a retrospective analysis. Second, the sample size of human participants is too small. Other confounders (like food intake and physical activity of participants) were not considered in the analysis.

## 5. Conclusion

Our study found that PCOS patients with combined Hashimoto’s thyroiditis may be more prone to disorders of glucose and lipid metabolism including increased insulin resistance and cholesterol levels. Thus, thyroid function and metabolic status in patients with PCOS should be evaluated in clinical practice.

## Acknowledgments

The authors express their gratitude to all of the participants who made this study possible, as well as the doctors and researchers who assisted them.

## Author contributions

**Data curation:** Cenlin Jia, Xiao Yan Zhang, Hongyan Wu.

**Methodology:** Wenhua Liu.

**Supervision:** Wenhua Liu.

**Writing – original draft:** Cenlin Jia.

**Writing – review & editing:** Cenlin Jia, Hongyan Wu, Lin Zhang.

## References

[1] Yang R Yang S Li R. Effects of hyperandrogenism on metabolic abnormalities in patients with polycystic ovary syndrome: a meta-analysis. Reprod Biol Endocrinol. 2016;14:67. 27756332 10.1186/s12958-016-0203-8 PMC5069996

[2] Abtahi-Eivari SH Moghimian M Soltani M. The effect of Galega officinalis on hormonal and metabolic profile in a rat model of polycystic ovary syndrome. Int J Women’s Health Reprod Sci. 2018;6:276–82.

[3] Khodaeifar F Fazljou SMB Khaki A. Investigating the role of hydroalcoholic extract of apium graveolens and cinnamon zeylanicum on metabolically change and ovarian oxidative injury in a rat model of polycystic ovary syndrome. Int J Women’s Health Reprod Sci. 2019;7:92–8.

[4] Cai J Zhang Y Wang Y. High thyroid stimulating hormone level is associated with hyperandrogenism in euthyroid polycystic ovary syndrome (PCOS) women, independent of age, BMI, and thyroid autoimmunity: a cross-sectional analysis. Front Endocrinol (Lausanne). 2019;10:222. 31024459 10.3389/fendo.2019.00222 PMC6467931

[5] Arduc A Aycicek Dogan B Bilmez S. High prevalence of Hashimoto’s thyroiditis in patients with polycystic ovary syndrome: does the imbalance between estradiol and progesterone play a role? Endocr Res. 2015;40:204–10. 25822940 10.3109/07435800.2015.1015730

[6] Gaberšček S Zaletel K Schwetz V. Mechanisms in endocrinology: thyroid and polycystic ovary syndrome. Eur J Endocrinol. 2015;172:R9–21. 25422352 10.1530/EJE-14-0295

[7] Detti L Jeffries-Boyd HE Williams LJ. Fertility biomarkers to estimate metabolic risks in women with polycystic ovary syndrome. J Assist Reprod Genet. 2015;32:1749–56. 26542956 10.1007/s10815-015-0602-3 PMC4681743

[8] Karimi E Heshmati J Shirzad N. The effect of synbiotics supplementation on anthropometric indicators and lipid profiles in women with polycystic ovary syndrome: a randomized controlled trial. Lipids Health Dis. 2020;19:60. 32248805 10.1186/s12944-020-01244-4 PMC7132870

[9] Heshmati J Sepidarkish M Morvaridzadeh M. The effect of cinnamon supplementation on glycemic control in women with polycystic ovary syndrome: a systematic review and meta-analysis. J Food Biochem. 2021;45. 10.1111/jfbc.13543 33111340

[10] Schwetz V Pieber T Obermayer-Pietsch B. Mechanisms in endocrinology: thyroid and polycystic ovary syndrome. Eur J Endocrinol. 2015;172:R9–R21. 25422352 10.1530/EJE-14-0295

[11] Liu L Jin Z Cai X. Comparative regimens of lipid rescue from bupivacaine-induced asystole in a rat model. Anesth Analg. 2019;128:256–63. 30113398 10.1213/ANE.0000000000003711

[12] Zhao H Zhang Y Ye J. A comparative study on insulin secretion, insulin resistance and thyroid function in patients with polycystic ovary syndrome with and without Hashimoto’s thyroiditis. Diabetes Metab Syndr Obes. 2021;14:1817–21. 33953581 10.2147/DMSO.S300015 PMC8089092

[13] Singh J Wong H Ahluwalia N. Metabolic, hormonal, immunologic, and genetic factors associated with the incidence of thyroid disorders in polycystic ovarian syndrome patients. Cureus. 2020;12:e11681. 33391917 10.7759/cureus.11681 PMC7769736

[14] Romitti M Fabris VC Ziegelmann PK. Association between PCOS and autoimmune thyroid disease: a systematic review and meta-analysis. Endocr Connect. 2018;7:1158–67. 30352422 10.1530/EC-18-0309 PMC6215798

[15] Singla R Gupta Y Khemani M. Thyroid disorders and polycystic ovary syndrome: an emerging relationship. Indian J Endocrinol Metab. 2015;19:25–9. 25593822 10.4103/2230-8210.146860 PMC4287775

[16] Pergialiotis V Konstantopoulos P Prodromidou A. Management of endocrine disease: the impact of subclinical hypothyroidism on anthropometric characteristics, lipid, glucose and hormonal profile of PCOS patients: a systematic review and meta-analysis. Eur J Endocrinol. 2017;176:R159–66. 28007842 10.1530/EJE-16-0611

[17] de Medeiros SF de Medeiros MAS Ormond CM. Subclinical hypothyroidism impact on the characteristics of patients with polycystic ovary syndrome. a meta-analysis of observational studies. Gynecol Obstet Invest. 2018;83:105–15. 30025406 10.1159/000485619

[18] Ho CW Chen HH Hsieh MC. Increased risk of polycystic ovary syndrome and it’s comorbidities in women with autoimmune thyroid disease. Int J Environ Res Public Health. 2020;17:2422. 32252386 10.3390/ijerph17072422 PMC7177418

